# Asymptomatic Late Migration of an Atrial Pacemaker Lead into the Right Lung

**DOI:** 10.1155/2014/145917

**Published:** 2014-11-12

**Authors:** Nicolas De Schryver, Sebastien Marchandise, Geoffrey C. Colin, Benoît Ghaye, Jean-Benoît le Polain de Waroux

**Affiliations:** ^1^Division of Cardiology, Unit of Electrophysiology and Cardiac Pacing, Cliniques Universitaires St-Luc, Université Catholique de Louvain, Avenue Hippocrate 10, 1200 Brussels, Belgium; ^2^Division of Radiology, Unit of Cardiac and Thoracic Imaging, Cliniques Universitaires St-Luc, Université Catholique de Louvain, Avenue Hippocrate 10, 1200 Brussels, Belgium

## Abstract

This report illustrates an unusual case of asymptomatic late cardiac perforation by an atrial pacemaker lead into the right lung. In the present case, the lead was explanted by simple manual traction through the device pocket without any complications.

## 1. Case Report

**Figure 1:**
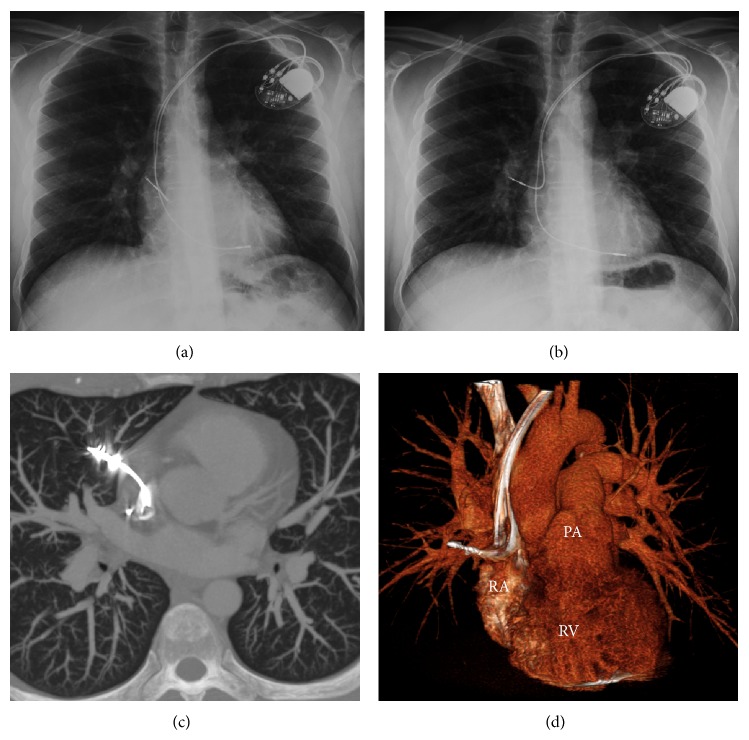
(a) Postimplant anteroposterior chest radiography showing the atrial lead positioned at the basal part of the right atrial appendage. (b) Anteroposterior chest radiography performed at 8 months showing a clear late migration of the atrial lead into the right lung. (c) Contrast-enhanced CT-scanner with maximal intensity projection (MIP) and (d) volume rendered reformatting (anterior view) demonstrating the migration of the atrial lead into the right lung. Notably the pericardium, pleura, and pulmonary parenchyma were normal. RA: right atrium; RV: right ventricle; PA: pulmonary artery.

A 33-year-old patient with no evidence of underlying cardiomyopathy was implanted with a dual-chamber pacemaker (St-Jude Medical (SJM, St. Paul, Minnesota, USA) Zephyr XL DR; active fixation atrial lead SJM Tendril ST 1882T/52; passive ventricular lead SJM Tendril ST 1888T/58) for symptomatic complete atrioventricular (AV) block. The implantation procedure was uneventful and usual follow-up including device interrogation and chest radiography remained normal until the 8-month follow-up visit. During this last consultation, although the patient remained totally asymptomatic, both atrial sensing and capture were lost. Chest radiography revealed a clear migration of the atrial lead into the right lung (Figure 1). Contrast-enhanced chest computed tomographic (CT) scanner confirmed the migration of the atrial lead through the right appendage, the pericardium, the pleurae, and finally the lung parenchyma. No pleural effusion or pneumothorax was observed. The extraction of the lead was performed in the operating room, under general anaesthesia and with transoesophageal echocardiographic monitoring. The lead was simply removed by pulling it manually gently out from the pacemaker pocket. The patient was discharged the next day and no further complication was observed during follow-up.

## 2. Discussion

Pacemaker implantation is a very common procedure that usually carries a low risk of complications. Acute perforation of a cardiac cavity by a pacemaker lead occurs in about 1% of the patients at time of the implantation [1, 2]. However, this rate could rise up to 10% when considering early occurrence of asymptomatic pericardial effusion [3]. Late myocardial perforation by a pacemaker lead (>1 month) is a very rare complication that has been described in only few case reports. Clinical presentation usually includes pleuritic chest pain, diaphragmatic stimulation, cough, or haemoptysis. In some rare cases, like in our patient, it remains totally asymptomatic. The management of a cardiac perforation by a pacemaker lead is challenging. The true risk of abandoning a lead in such position is unknown. However, as severe mechanical complications (tamponade, haemothorax, etc.) may occur, it seems safer to explant the lead. In the literature, most of the cases of late myocardial perforation were managed by surgical removal of the lead via an open thoracotomy. In the present case, however, it was removed by simply manually pulling out the lead through the device pocket. Although the risk might not be negligible, it seems justified in our opinion to try first to simply remove the lead through the implantation site. Nevertheless, we consider it safer to perform this procedure under general anaesthesia, with a transoesophageal monitoring and with a surgical backup.

## 3. Conclusion

This case report illustrates a rare case of asymptomatic late cardiac perforation of an atrial pacemaker lead into the right lung. In such situation, simple manual lead extraction through the device pocket is worth trying and allows for avoiding an invasive surgical approach.
